# Factors impacting trial participation in people with motor neuron disease

**DOI:** 10.1007/s00415-023-12010-8

**Published:** 2023-10-03

**Authors:** Emily Beswick, Micheala Johnson, Judith Newton, Rachel Dakin, Amy Stenson, Sharon Abrahams, Alan Carson, Siddharthan Chandran, Suvankar Pal

**Affiliations:** 1 Centre for Clinical Brain Sciences, The University of Edinburgh, Edinburgh, Scotland; 2 Anne Rowling Regenerative Neurology Clinic, The University of Edinburgh, 49 Little France Crescent, Edinburgh, EH16 4 SB Scotland; 3 Euan MacDonald Centre for MND Research, The University of Edinburgh, Edinburgh, Scotland; 4 Human Cognitive Neurosciences, Psychology, School of Philosophy, Psychology and Language Sciences, The University of Edinburgh, Edinburgh, Scotland; 5 UK Dementia Research Institute, The University of Edinburgh, Edinburgh, Scotland

**Keywords:** Motor neuron disease, Amyotrophic lateral sclerosis, Clinical trials, Recruitment

## Abstract

Motor neuron disease (MND) is a rapidly progressive neurodegenerative disorder with limited treatment options. Historically, neurological trials have been plagued by suboptimal recruitment and high rates of attrition. The Motor Neuron Disease–Systematic Multi-Arm Randomised Adaptive Trial (MND–SMART) seeks to identify effective disease modifying drugs. This study investigates person-specific factors affecting recruitment and retention. Improved understanding of these factors may improve trial protocol design, optimise recruitment and retention. Participants with MND completed questionnaires and this was supplemented with clinical data. 12 months after completing the questionnaires we used MND–SMART recruitment data to establish if members of our cohort engaged with the trial. 120 people with MND completed questionnaires for this study. Mean age at participation was 66 (SD = 9), 14% (*n = *17) were categorised as long survivors, with 68% (*n = *81) of participants male and 60% (*n = *73) had the ALS sub-type. Of the 120 study participants, 50% (*n = *60) were randomised into MND–SMART and 78% (*n = *94) expressed interest an in participating. After the 1-year follow-up period 65% (*n = *39) of the 60 randomised participants remained in MND–SMART. Older age was significantly associated with reduced likelihood of participation (OR = 0.92, 95% CI = 0.88–0.96, *p = *0.000488). The findings show that people with MND are highly motivated to engage in research, but older individuals remain significantly less likely to participate. We recommend the inclusion of studies to explore characteristics of prospective and current participants alongside trials.

**Supplementary Information:**

The online version contains supplementary material available at 10.1007/s00415-023-12010-8.

## Introduction

### Motor neuron disease

Motor neuron disease (MND) is a devastating progressive neurological disorder, affecting multiple aspects of functioning and ultimately resulting in death from respiratory failure [1]. The global prevalence of MND is reported as 4.42 (95% CI 3.92–4.96) per 1,00,000 population and incidence of 1.59 (95% CI 1.39–1.81) per 1,00,000 person-years [2].

### Trials in MND

Despite 125 clinical trials registered between 2008 and 2019, involving 15,647 people with MND and evaluating 76 investigative medicinal products (IMPs) [3], progress in developing new treatments has been underwhelming [4]. However, in the last decade new directions in MND trials are emerging. Advanced understanding of the biological basis of the condition, novel biomarkers and multiple potential therapeutic targets offer promising avenues of exploration [5]. The impact of Edaravone [6] and AMX0035 [7] for some people with MND, resulted in their approval by the Food and Drug Association (FDA), and is under investigation in a European cohort (NCT05178810). The FDA has recently approved toferson in people with a superoxide dismutase (SOD1)-associated amyotrophic lateral sclerosis. Toferson was reported to be most effective if started earlier in the disease course. The ATLAS trial is investigating the impact on pre-symptomatic SOD1 mutation carriers (NCT02623699). Whilst such encouraging advances are being made with targeted genetic therapies, there is major unmet need for more efficacious treatments in sporadic MND which affect the majority of individuals.

MND–SMART (Motor Neuron Disease–Systematic Multi-Arm Randomised Adaptive Trial) is a phase 3 UK-wide innovative adaptive platform trial, clinicaltrials.gov (NCT04302870) and EudraCT (Trial record number: 2019-000099-41) recruiting people with a clinical diagnosis of MND, irrespective of genetic status.

MND–SMART aims to evaluate a series of drugs over the next two decades, within an adaptive platform trial protocol. Adaptive design trials use the ongoing results of the trial to feedback recommendations on the continuation or early stopping of candidate drugs, with the platform aspect ensuring new candidate drugs can be introduced during the course of the trial. All IMPs are administered in a liquid form, to ensure participants can continue to take them if difficulties with swallowing develop or progress. Broad inclusion criteria promotes wide participation and ensures that large numbers of people living with MND are eligible, with no sub-type exclusions to capture disease heterogeneity and improve the generalisability of findings.

### Recruitment and retention

Successful recruitment to clinical trials requires engagement of participants representative of the wider target population, in numbers sufficient to meet the requirements of trial-specific power calculations, in efficient time-frames [8]. Minimising attrition is also an important consideration in trial design. Clinical trials in MND frequently report attrition rates over 20% [9, 10], a threshold where risk of bias is high [11].

Sub-optimal recruitment and retention can affect the ability to draw valid conclusions from trial data, and increasing the probability of Type II error [6]. Trials that do not recruit efficiently, can have accrue high levels of financial cost, and contribute to wastage if participant time and data [12, 13]. The accurate identification of factors that impact upon recruitment and retention of participants in research studies is essential to informing trial design [12].

The current study is the first to prospectively explore the characteristics of a group of people with MND, in parallel with the launch of a national trial, and follow up on their involvement after a one year time frame. In these findings we report the demographics, clinical features and attitudes to research of a subgroup of people with MND who were in the process of decision-making, related to an actively recruiting trial, and the outcome of this decision.

### Aims

The general aim of this study was to investigate person-specific factors affecting recruitment and retention of people with MND to MND–SMART.

Specifically, this study aimed to:

1. Explore the clinical and demographic characteristics of participants who choose to participate in MND–SMART, in comparison with those who do not become involved in the trial.
2. Explore the clinical and demographic characteristics of study participants who were also MND–SMART participants, but were not retained one year after study participation, compared to those who remained trial participants.

### Hypothesis

We hypothesise that person-specific factors, such as neuropsychiatric symptoms, cognitive impairment, behavioural change, phenotype, quality of life, apathy and physical functioning will significantly impact upon people with MND’s decision to participate, and remain in MND–SMART.

## Materials and methods

The full method for this study, power calculations, assessment selection and analysis plan are outlined in the protocol paper [14], with a summary provided here and study questionnaires included as an Appendix.

The Scottish MND Register (Clinical Audit Research and Evaluation of MND–CARE–MND) has a strong history of high case ascertainment associated with longitudinal clinical phenotyping, collating many of the clinical variables included in this study. CARE–MND is also a register of people with MND who are interested in research participation and was used to facilitate recruitment [15]. As the current study relied on a Scottish-wide register for data and recruitment, this study focused on recruitment and retention in participants at MND–SMART sites in Scotland.

Data requests to CARE–MND and MND–SMART for information relating to physical functioning, cognition and clinical phenotype enabled a reduction in in burden for participants by ensuring brevity in study visits. Data on MND–SMART participation was requested a minimum of 12 months after study questionnaires were completed. This study recruited 120 individuals with a diagnosis of MND. The sample size calculation was based upon the use of a logistical regression model, recruitment to MND–SMART clinical trial is a binary outcome variable (Yes/No to participation). An OR (measure of association between an exposure and an outcome) of 1.70 with power at 0.70 provided a sample size estimate of 111.

### Study assessments

Participant and caregiver questionnaire data were supplemented with data derived from CARE–MND or MND–SMART relating to cognition (Edinburgh Cognitive and Behavioural Amyotrophic Lateral Sclerosis Screen (ECAS) [16]), disease phenotype, demographics and physical functioning (ALSFRS(R)) and the full questionnaire schedule is available in the appendices. Participants were also invited to completed the Attitudes towards Clinical Trial Participation Questionnaire (ACT-Q), developed specifically for this study. The ACT-Q involved 19 5-point Likert rating scales on agreement or understanding of statements related to barriers or reasons for research participation, and understanding of clinical trials.

The study involved three stages of data collection:

1. Questionnaire completion: participants and caregivers complete questionnaires
2. CARE–MND data request: additional covariates collected in routine clinical care
3. MND–SMART data request: trial involvement and participation

### Analysis plan

Each potential response on the ACT-Q was scored on the participant’s rating of its importance to their decision making process and the mean score for each aspect was be represented.

The independent variables in this study were the study questionnaires and clinical data from CARE–MND. The binary dependent variable was the decision to participate, or not, in MND–SMART. To determine which factors affected recruitment into the trial, logistic regression was used to model aforementioned independent variables on the dependent variable. Results were presented with odds ratio and 95% confidence intervals.

To determine which factors affected trial retention, we used univariate and multivariable logistic regression to explore the effect of the independent variables on withdrawal from the trial at the 12-month timepoint (dependent variable). Results were displayed as odds ratios and 95% confidence intervals.

## Results

### Participant overview

Between 20th August 2020 and 27th May 2021, 120 people with MND residing in Scotland participated in this observational cohort study. A total of 374 people with MND on the CARE–MND register were invited to participate, 158 (42%) completed consent forms and 120 (32%) went on to participate in the current study. The baseline characteristics of participants are displayed in Table 1 and generally representative of the heterogeneity found in MND [17].

**Table 1:** Characteristics of study participants and CARE–MND participants

Characteristics	Study participants (*N = *120)	Consent to Share clinical data for research provided on CARE–MND register (*N = *295)	Consent to be contact about research studies provided on CARE–MND register (*N = *216)
Age at participation in years, mean (SD)	66 (9)	N/A	N/A
Age at diagnosis in years, mean (SD)	62 (10)	61 (14)	60 (14)
Survival length (%)			
Long survivors (> 7 years)	17 (14)	55 (19)	39 (18)
Males, no. (%)	81 (68)	172 (58)	136 (63)
MND sub-type, no. (%)			
ALS	73 (60)	193 (65)	135 (63)
PLS	16 (13)	31 (11)	25 (12)
PBP	15 (12)	25 (8)	18 (8)
PMA	5 (4)	10 (3)	9 (4)
Other	3 (3)	11 (4)	8 (3)
No data	8 (8)	25 (9)	21 (10)
Bulbar onset (%)	28 (23)	89 (30)	61 (28)
Intervention use (%)			
Riluzole	39 (33)	91 (31)	74 (34)
Non-invasive ventilation	12 (10)	47 (16)	40 (19)
Gastrostomy	19 (16)	67 (23)	45 (21)

*SD* standard deviation, *CARE–MND* Clinical Audit Research and Evaluation Register, *N/A* not applicable, *MND* motor neurone disease, *ALS* amyotrophic lateral sclerosis, *PLS* primary lateral sclerosis, *PBP* progressive bulbar palsy, *PMA* progressive muscular atrophy

Also displayed in Table 1 for comparative purposes, are the characteristics of all individuals on CARE–MND who did not participate in the current study but have agreed to share their clinical data for research, ‘CARE–MND Data Consent’ (*N = *295). Within this group of individuals who had consented to share their data, 73% (*N = *216) had provided additional consent to be contacted about participating in research. These 216 individuals did not participate in, or complete consent forms for, the current study and their characteristics are presented in an additional column.

### Attitudes to trials questionnaire

The ACT-Q explored three areas of interest in trial engagement; potential barriers to participation, reasons for participation and understanding of clinical trial design. Mean scores and associated interpretations for each item are displayed in Table 2.

**Table 2:** Responses to the ACT-Q

	Mean score, interpretation (*n*)
Barriers to participation^c^	
(2) The distance to the clinic is too far	3.2, Slightly important (118)
(1) The time commitment required to participate	2.9, Slightly important (118)
(6) I am concerned about the potential dangers and side effects of trial medications^b^	2.9, Slightly important (119)
(7) I am worried about the possibility of being assigned to the placebo group^b^	2.7, Slightly important (117)
(3) I would not feel well enough to participate because of how my condition affects me	2.6, Slightly important (119)
(5) I may not benefit personally from the development of new drugs	2.4, Not at all (117)
(4) I am already participating in other research projects	2.3, Not at all (116)
(8) I already feel I have a lot of appointments	2.2, Not at all (118)
Reasons for participation^c^	
(12) I want to help other people with the same condition as me	4.8, Very important (118)
(14) I want the opportunity to contribute to research	4.6, Very important (119)
(9) I may get to try new medicines which are not available to everyone with my condition	4.1, Quite important (119)
(10) I will get additional monitoring of how my condition is changing	3.9, Quite important (119)
(11) I will receive more regular contact with medical staff	3.7, Quite important (118)
(13) I have participated in research before and had a positive experience^b^	2.4, Slightly important (116)
Understanding of clinical trials and multi-arm multi-stage design^a^	
(15) Why clinical trials often need to have a group of people taking a placebo drug	4.0, Good understanding (119)
(16) Why not everyone may be suitable to participate in a specific clinical trial	4.0, Good understanding (118)
(17) Why researchers believe these experimental treatments may be effective	3.9, Good understanding (119)
(19) Why having multiple ‘arms’ means we can test more than one drug and why this matters	3.5, Some understanding, (119)
(18) What is a re-purposed medicine and why may it be effective in my condition	3.4, Some understanding (119)

^c^1 = Do not know, 2 = Not at all, 3 = Slightly important, 4 = Quite important, 5 = Very important

^b^1 = No understanding, 2 = Little understanding, 3 = Some understanding, 4 = Good understanding, 5 = Full understanding

^a^Items have been reverse scored, indicating less agreement with the attitude

Barriers and reasons for participation are ranked in order of their reported importance to participants. Key barriers to participation identified were; the distance to the clinic, dangers and side effects from trial medications and the time commitment required to participate. Key reasons for participation identified were; wanting to help others with the same condition, the opportunity to contribute to research and the possibility of trying new medications that are not available to all people with MND, increased contact with medical team.

Participants’ understanding of clinical trials, and the complexities of multi-arm multi-stage design are ranked from best understood to least understood. The best understood areas were necessity of placebos, exclusion criteria and potential treatment efficacy, whilst use of multi-arm design and re-purposed medicines were rated as less well-understood.

### Overview of recruitment and retention

120 people with MND completed the questionnaires and assessments. Follow-up data on trial engagement was collected 12 months from the completion of the final questionnaire. Each participant was followed up for a mean of 18 months (SD = 2.5) after completing their questionnaires, with a minimum of 12 months for each individual. A total of 47 participants died during the follow-up period (overall 12-month survival of 61%).

Of these individuals, 26 people did not complete online interest forms, or contact the trial team, about potentially participating in MND–SMART and this group was labelled ‘No Interest Expressed’. The remaining 94 (78%) expressed some form of interest in engaging, termed ‘Interest Expressed’; and 60 (50%) of them went on to be randomised into the trial, termed ‘Randomised’. 22 people changed their mind, died or progressed too quickly to become involved, a group labelled ‘No Participation’, and 12 were unable to join due to not meeting inclusion criteria when screened by the trial team, the ‘Screen Failure’ group.

After the 1-year period, data on trial engagement was evaluated. Of the 60 study participants who were randomised into MND–SMART, 39 (65%) remained a participant at the 1-year data collection timepoint, in the ‘Remain Participant’ group. Of the 21 individuals who were no longer participants this was primarily due to death, with only 1 person being withdrawn from the trial, the remaining 20 people had died, included in the ‘Previous Participant’ group.

Table 3 provides the number of individuals, and their mean scores for study questionnaires and assessments, for each of the trial participation groups.

**Table 3:** Demographics, clinical features and assessment scores per trial group

	Interest in participation shown	Remaining in MND–SMART participants	MND–SMART participants	No engagement
	Registered interest with the trial team, were screened for participation or joined MND–SMART	Participated in MND–SMART and remain participants at 1-year timepoint	Participated in MND–SMART at some stage in the 1-year time frame	Did not become MND–SMART participants and did not register interest
	*n = *93	*n = *38	*n = *59	*n = *48
Gender				
Male	63	25	41	31
Female	30	13	18	17
Long survivor				
Yes	19	10	11	15
No	74	28	48	33
Region of onset				
Lower limb	37	20	25	16
Upper limb	21	10	14	9
Bulbar	18	5	13	16
Respiratory	2	2	2	-
Mixed	9	1	3	5
No data	6	–	2	2
Sub-type				
ALS	59	24	36	33
PLS	14	8	9	5
PMA	3	2	2	2
PBP	9	2	8	6
MND–FTD	1	–	–	1
Other	2	1	2	–

	Mean (SD)			
Age	64.91 (9.01)	62.66 (10.29)	63.53 (9.35)	70 (7.98)
Number of interventions used	0.56 (0.63)	0.53 (0.60)	0.63 (0.64)	0.65 (0.73)
Number of studies previously participated in	0.91 (1.39)	1.13 (1.38)	1.02 (1.47)	1.02 (1.45)
ECAS total score	109.73 (13.32)	108.97 (12.85)	109.48 (13.23)	116.17 (10.74)
ALSFRS (R)	32.99 (8.87)	34.58 (7.63)	32.72 (9.02)	30.53 (9.59)
HADS				
Anxiety	5.99 (3.91)	5.79 (4.13)	6.31 (4.20)	5.58 (4.01)
Depression	6.16 (3.56)	5.32 (3.29)	6.00 (3.62)	6.92 (3.04)
Total	12.44 (6.85)	11.34 (7.14)	12.56 (7.31)	12.71 (6.53)
STAI				
State	45.84 (5.54)	46.50 (5.34)	45.92 (5.73)	45.44 (6.25)
Trait	45.24 (5.22)	45.76 (5.13)	45.25 (5.30)	43.65 (6.68)
Total	90.87 (9.44)	92.05 (8.92)	91.03 (9.62)	88.65 (11.05)
PHQ				
Total	6.86 (5.29)	6.89 (4.14)	7.02 (4.55)	7.17 (6.03)
Presence of suicidality *n* (%)	18 (19)	4 (11)	11 (19)	11 (23)
ALSQOL	4.80 (1.39)	4.74 (1.46)	4.98 (1.39)	4.96 (1.41)
HRQOL				
Overall health	2.86 (1.12)	2.95 (1.06)	2.93 (1.08)	2.46 (1.07)
Physical health symptoms	13.87 (12.86)	14.76 (12.79)	13.14 (12.70)	18.62 (13.15)
Mental health symptoms	7.17 (9.49)	9.38 (11.16)	8.60 (10.42)	5.48 (7.82)
Days impacting usual activities	9.24 (12.25)	10.03 (12.57)	10.28 (12.89)	10.75 (12.71)
bDAS	7.82 (5.27)	6.26 (4.70)	6.77 (4.89)	8.45 (5.21)

HRQOL ratings of overall health options are; 5 = Excellent, 4 = Very Good, 3 = Good, 2 = Fair, or 1 = Poor

*SD* standard deviation, *MND–SMART* motor neuron disease–systematic multi-arm randomised adaptive trial, *MND* motor neurone disease, *ALS* amyotrophic lateral sclerosis, *PLS* primary lateral sclerosis, *PBP* progressive bulbar palsy, *PMA* progressive muscular atrophy, *MND–FTD* motor neurone disease–frontotemporal dementia, *ECAS* Edinburgh Cognitive and Behavioural Amyotrophic Lateral Sclerosis Screen, *ALSFRS-R* amyotrophic lateral sclerosis functional rating scale revised, *HADS* hospital anxiety and depression scale, *STAI* state–trait anxiety inventory, *PHQ* patient health questionnaire, *ALSQOL* amyotrophic lateral sclerosis quality of life, *HRQOL* health related quality of life, *bDAS* brief dimensional apathy scale

### Variables associated with recruitment to MND–SMART

Tables 4 and 5 provide full detail on the Chi-square tests and logistic regressions used to explore recruitment. Age was a significant predictor for trial participation, (OR = 0.92, 95% CI = 0.88–0.96, *p = *0.000488), indicating that for every increase in 1 year of age, the odds of participating in MND–SMART decreased by 1%.

**Table 4:** Logistic regressions to explore recruitment and retention to MND–SMART

	Estimate	OR	95% CI	*z*	*df*	*p* value
Recruitment						
Intercept						
Number of studies	− 0.57			− 0.26	117	0.81
Previously participated in	0.04	1.04	0.80–1.35	0.32		0.75
Intercept	5.46			3.45	117	0.0006**
Age at participation	− 0.08	0.92	0.88–0.96	− 3.49		0.0005**
Intercept	− 0.12			− 0.49	117	0.63
Number of interventions	0.17	1.19	0.69–2.06	0.63		0.53
Intercept	3.90			1.41	65	0.16
ECAS	− 0.02	0.98	0.93–1.02	− 0.97		0.33
Intercept	− 0.122			− 0.17	105	0.86
ALSFRS(R)	0.009	1.01	0.97–1.05	0.42		0.67
Intercept					117	
HADS total	− 0.15	1.01	0.96–1.06	− 0.04		0.69
HADS anxiety	0.01	1.06	0.97–1.17	0.40		0.68
HADS depression	0.06	0.96	0.87–1.07	1.32		0.18
Intercept	− 1.10			− 0.68	117	0.50
STAI total	0.01	1.01	0.98–1.05	0.67		0.50
STAI state	0.001	1.001	0.94–1.06	0.03		0.98
STAI trait	0.03	1.03	0.97–1.10	0.94		0.35
Intercept	− 0.16			− 0.52	117	0.60
PHQ total	0.02	1.02	0.95–1.10	0.58		0.56
Intercept	− 0.54			− 0.84	117	0.40
ALSQOL	0.11	1.12	0.88–1.45	0.84		0.40
Intercept	0.69			1.67	84	0.11
bDAS	− 0.08	0.92	0.84–1.00	− 1.82		0.07
Retention						
Intercept						
Number of studies	0.87			− 3.60		0.0003***
Previously participated in	0.11	1.11	0.85–1.45	0.82	117	0.41
Intercept	4.13			2.62		0.009**
Age at participation	− 0.07	0.93	0.88–0.97	− 3.09	117	0.002**
Intercept	− 0.64			− 2.47	117	0.01*
Number of interventions	− 0.21	0.81	0.44–1.45	− 0.69		0.49
Intercept	2.17			1.01		0.31
ECAS	0.02	0.98	0.94–1.02	− 0.92	65	0.36
Intercept	− 2.05			− 2.50		0.01*
ALSFRS(R)	0.04	1.05	1.01–1.10	1.82	105	0.07
Intercept	-0.37			− 0.91	117	0.36
HADS total	− 0.03	0.97	0.90–1.03	− 1.05		0.29
HADS anxiety	− 0.002	1.10	0.90–1.09	− 0.05		0.96
HADS depression	− 0.12	0.89	0.78–1.10	− 1.93		0.05
Intercept	− 2.9			− 1.59	117	0.11
STAI total	0.02	1.02	0.99–1.07	1.19		0.23
STAI state	0.03	1.03	0.96–1.10	0.76		0.45
STAI trait	0.04	1.05	0.98–1.12	1.28		0.20
Intercept	− 0.81			− 2.51	117	0.01*
PHQ total	0.009	1.01	0.93–1.09	0.23		0.82
Intercept	− 0.35			− 0.51	117	0.61
ALSQOL	− 0.08	0.92	0.70–1.20	− 0.61		0.54
Intercept	0.19			0.45		0.65
bDAS	− 0.10	0.90	0.81–0.99	− 2.01	84	0.04*

*Indicates significance at *p < *0.05

**Indicates significance at *p < *0.01

***Indicates significance at *p < *0.00

**Table 5:** Chi-square tests explore recruitment and retention to MND–SMART

	*N*	*X*^2^	*df*	*p* value
Recruitment				
Gender	119	0.02	1	0.89
Long survivor	119	0.68	1	0.41
Region of onset	112	7.81	5	0.17
Disease subtype	115	4.76	6	0.57
Caregiver co-participating	119	0.12	1	0.72
PHQ suicidality presence	119	5.2	1	1.00
Retention				
Gender	119	0.03	1	0.88
Long survivor	119	0.17	1	0.68
Region of onset	112	17.79	5	0.003**
Disease subtype	115	6.52	6	0.37
Caregiver Co-participating	119	1.78	1	0.18
PHQ suicidality presence	119	1.64	1	0.2

**Indicates significance at *p < *0.01

Region of onset, disease subtype, long survivor status or number of life-prolonging interventions used were not associated with participation in MND–SMART. The number of studies previously participated in, co-participation with a caregiver and the presence of suicidality as indicated by PHQ response were also not associated. Scores on the ECAS, ALSFRS(R), HADS (total, depression and anxiety sub scores), STAI (total, state and trait sub scores), PHQ Total, ALSQOL and bDAS were not associated with participation in MND–SMART.

### Variables associated with retention to MND–SMART

In our study sample, of the 21 individuals who were no longer MND–SMART participants at the 1-year timepoint, only 1 individual withdrew from the trial and the other 20 remained trial participants until their death. As only 1 individual had withdrawn, there was an insufficient sample size for statistical analyses.

Instead we explored the characteristics of those who died during follow-up. Age of the participant was associated with them remaining in MND–SMART at the 1-year data collection timepoint, (OR = 0.93, 95% CI = 0.88–0.97, *p = *0.002). For every increase in 1 year of age, the odds of remaining a participant in MND–SMART decreased by 1%. In addition, bDAS scores were significantly associated with remaining in MND–SMART, (OR = 0.9, 95% CI = 0.80–0.99, *p = *0.044), indicating that for each one point increase in apathy severity score the odds of remaining a participant in MND–SMART decreased by 1.1%. No other clinical variables or assessment scores were associated with the likelihood of remaining a participant in MND–SMART or death during follow-up.

Region of onset was associated with remaining a participant in MND–SMART, *X*^2^ (5, *N = *112) = 17.79, *p = *0.003, with individuals with ‘Mixed’ symptom onset least likely to remain as an MND–SMART participant at the 1-year timepoint and more likely to die during the follow-up period.

## Discussion

### Study summary

The purpose of this study was to gain a better understanding of the person-specific factors that may influence an individual with MND’s decision to participate in a clinical trial and remain in the trial.

Of the 120 participants, 50% went on to be randomised to MND–SMART. An even greater number, 78%, of participants expressed interest in the trial (through completing online interest forms, contacting the trial team or attending screening appointments). Of the 60 study participants who were randomised to MND–SMART, 65% remained trial participants at the 1-year timepoint. Only one individual chose to withdraw from the trial, the remaining individuals who were not retained, remained trial participants until their death.

Participants in MND–SMART were highly motivated to engage with MND–SMART, and when randomised as trial participants remained involved with MND–SMART.

### Key findings

As in our study, previous findings have repeatedly shown that people with MND are highly motivated to engage with research [18]. Yet systematic reviews of previous trials indicate that only 25% are trial participants [19] and 40% of trials report > 20% attrition [3]. In this retrospective analysis the association between age, sex, race, disease duration, familial status, forced vital capacity and ASLFRS(R) and recruitment and retention was explored, however the reliance on existing data restricts the number of participant-specific variables available and limits analysis to people who were trial participants [18].

This suggests that prospective research, involving both current and potential participants, is crucial. Focusing on the identification and removal of barriers to participation and retention must remain a priority across MND.

This study indicated that age was a significant predictor for the likelihood of recruitment and retention to MND–SMART, with older individuals less likely to participate, and remain participating, in the trial. Reduced likelihood of participation from older age groups has also been identified as a concern in previous research [20], with older age used as an exclusion criteria for participation in some trials [21].

Increased severity of apathy, as indicated by caregiver responses to the b-DAS, was associated with reduced likelihood of retention to MND–SMART. The availability of literature exploring how apathy may impact research engagement was limited, with a focus on defining and measuring apathy [22], and recommendations for designing trials to evaluate interventions to target apathy [23].

Region of onset was the only clinical characteristic found to be associated with reduced retention rate to MND–SMART; individuals with a Mixed onset least likely to be retained, followed by those with Bulbar, then Upper and Lower Limb. Lower retention rates for people with Mixed and Bulbar onset was as expected, as these regions of onset are repeatedly linked to speech or swallow problems, and more rapid progression and worse prognosis than limb onset [24].

The finding that ALSFRS(R) scores were not significantly linked to trial engagement is inconsistent with previous study findings which showed better functionality at randomisation was a predictor of remaining a trial participant [18]. However, this lack of association may be influenced by our participants’ higher functional ability at the point of questionnaire completion. Study participants had a mean ALSFRS(R) total score of 32.5 (SD = 9.1), compared to studies with larger sample sizes, reporting a mean of 26.5 (SD = 10.3).

### Strengths of the study

This is the first prospective study exploring the characteristic of participants, and non-participants, in an MND trial. This study was conducted in parallel with the launch of a national multi-arm, multi-stage trial for repurposed candidate drugs, MND–SMART. The timeline was a key strength of this study, as we were able to evaluate participant-specific factors and attitudes as individuals were to be imminently faced with the possibility of trial participation.

Validated questionnaires on neuropsychiatric symptoms, behavioural change and quality of life were combined with clinical, demographic, cognitive and physical functioning data to provide an extensive list of participant-specific variables. In addition, attitudes towards participation and understanding of trial design, were explored in a questionnaire designed with the input of people with MND, specifically for this study.

### Future work

A key future direction in this area of research is increasing the frequency that person-specific factors and trial engagement are explored. Incorporating studies with a concept similar to the current study, either alongside trials, as studies-within-a-trial or sub-studies, can enable trialists to evaluate the characteristics of their own participants. Data on demographics, functional ability and clinical characteristics are routinely collected throughout an individuals’ trial journey, and secondary outcome measures focusing on neuropsychiatric, cognitive and behavioural symptoms are becoming more prevalent in trial design [3, 25] and can be used to evaluate secondary research aims.

Expanding on the variables explored in the current study may be a useful direction for future research, considering how broader social and demographic characteristics of prospective participants are associated with the decision to participate in a clinical trial. Socio-economic status, preferred language, marital status and education are potential areas to consider as impactful on trial participation. Genetic status is also a key variable to consider including in future research in this area, where genetic data are available, as awareness of genetic status may have an impact on individual’s decision to engage with a clinical trial.

For future research in this area it may be beneficial to explore how these person-specific factors affecting trial engagement decisions interact with trial design factors. MND–SMART uses repurposed drugs with known safety profiles, minimally invasive outcome measures and oral IMP administration. Exploring how person-specific factors affect the decision to enter into other trials which may have a higher participant burden, will be a key future direction to understand the decision process.

Future studies may also seek to overcome the sampling bias occurring in this study, which involved a cohort who self-selected as participants in a non-interventional study and were already more engaged in research than the wider MND population in Scotland. This may be done through exploring the characteristics of the sub-group who opted not to engage in any research participation, using data collected in routine clinical care, where people have provided permission for this information to be used in research, without a need to rely on active participation.

### Conclusion

Our research exemplifies the new, participant-centric, direction in delivering clinical trials outlined in the Airlie House Guidelines [26]. Understanding the characteristics of individuals who actively choose to participate in such trials enables trialists to make informed, and participant-focused decisions, about design, recruitment methods and retention strategies. Ultimately, this may have a positive impact in supporting more people with MND to engage with, and remain in, trials if they wish to do so.

The lack of association between person-specific variables and trial participation found in our study may suggest that engagement with a clinical trial is also dependent on design factors. Even when many of the design barriers raised by MND–SMART patient–public representatives were addressed, or minimised, in MND–SMART some sub-groups of people remained less likely to participate, and at a higher risk of attrition.

Further work to establish how individuals in these sub-groups may benefit from tailored interventions to support participation, and facilitate retention, is needed to effectively recruit and retain those currently under-represented in trial outcome data.

### Supplementary Information

Below is the link to the electronic supplementary material.

Supplementary file1 (XLSX 131 KB)

Supplementary file2 (DOCX 908 KB)

## Data Availability

Data are available as a supplementary file to this manuscript, with identifying information removed.
