# Supplementary material for: Factors impacting trial participation in people with motor neuron disease
Source: J Neurol. 2023 Oct 3;271(1):543–52. doi: 10.1007/s00415-023-12010-8 (PMC10769905; doi:10.1007/s00415-023-12010-8)

Factors Impacting Trial Participation in People with Motor Neuron Disease

**Appendices: Study Assessments**

Journal of Neurology

Emily Beswick^1,2,3^, Kay Johnson^1,2,3^, Judith Newton^1,2,3^, Rachel Dakin^1,2,3^, Amy Stenson^1,2,3^, Sharon Abrahams^3,4^, Alan Carson^1,2^, Siddharthan Chandran^1,2,3,5^ and Suvankar Pal^1,2,3^

1. Centre for Clinical Brain Sciences, The University of Edinburgh, Edinburgh, Scotland.
2. Anne Rowling Regenerative Neurology Clinic, The University of Edinburgh, Edinburgh, Scotland.
3. Euan MacDonald Centre for MND Research, The University of Edinburgh, Edinburgh, Scotland.
4. Human Cognitive Neurosciences, Psychology, School of Philosophy, Psychology and Language Sciences, the University of Edinburgh, Edinburgh, Scotland.
5. UK Dementia Research Institute, The University of Edinburgh, Edinburgh, Scotland.

**Corresponding author:**

Dr Suvankar Pal

Anne Rowling Regenerative Neurology Clinic

49 Little France Crescent

Edinburgh

EH16 4 SB

United Kingdom

E-Mail: Suvankar.Pal@ed.ac.uk


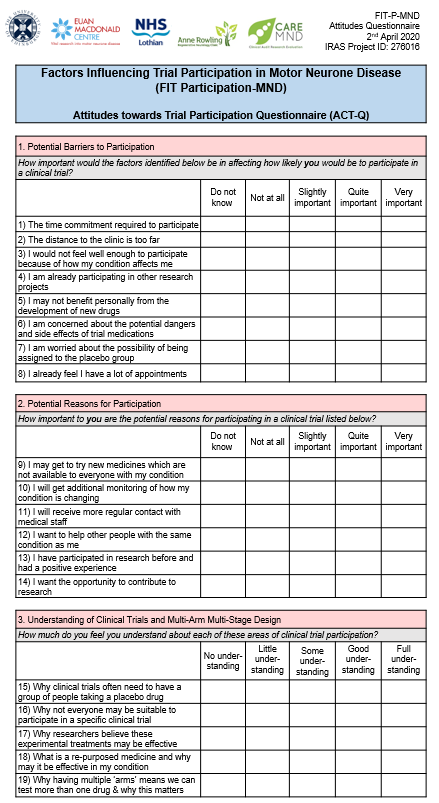


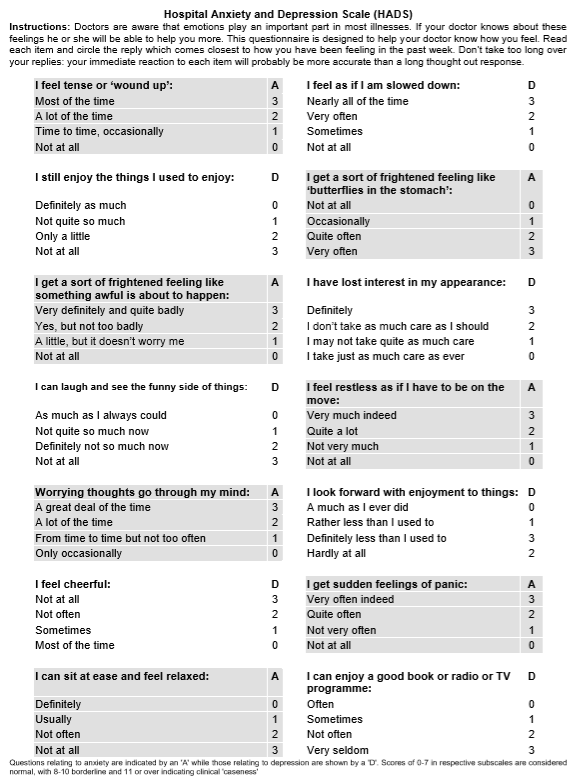


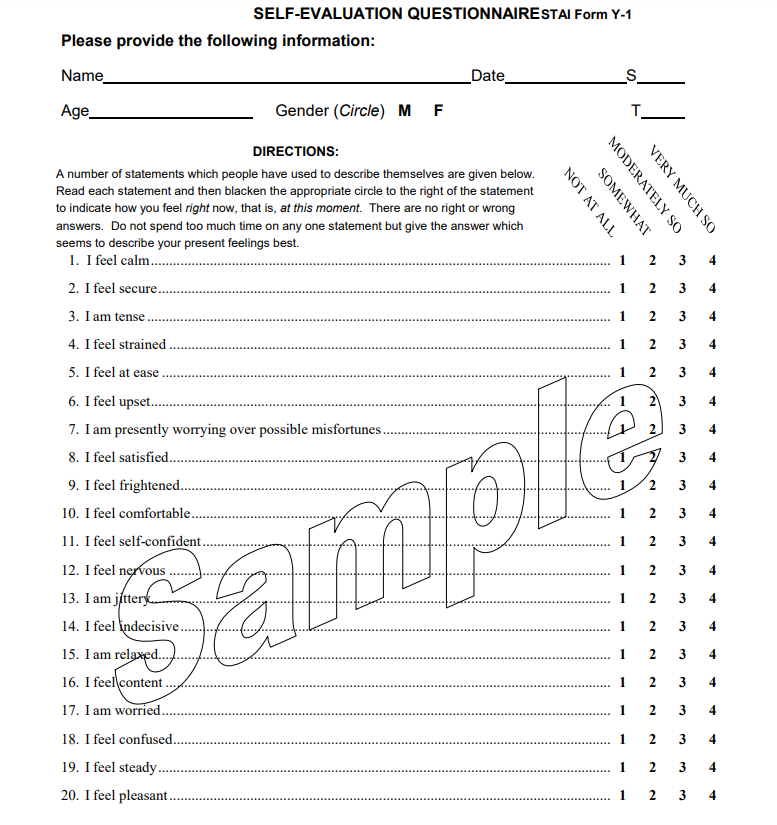


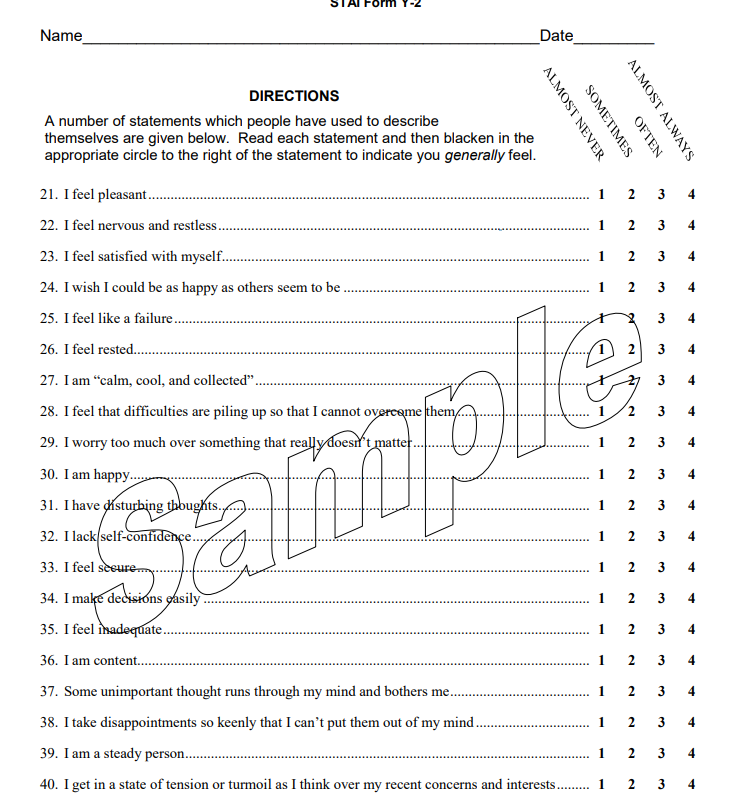


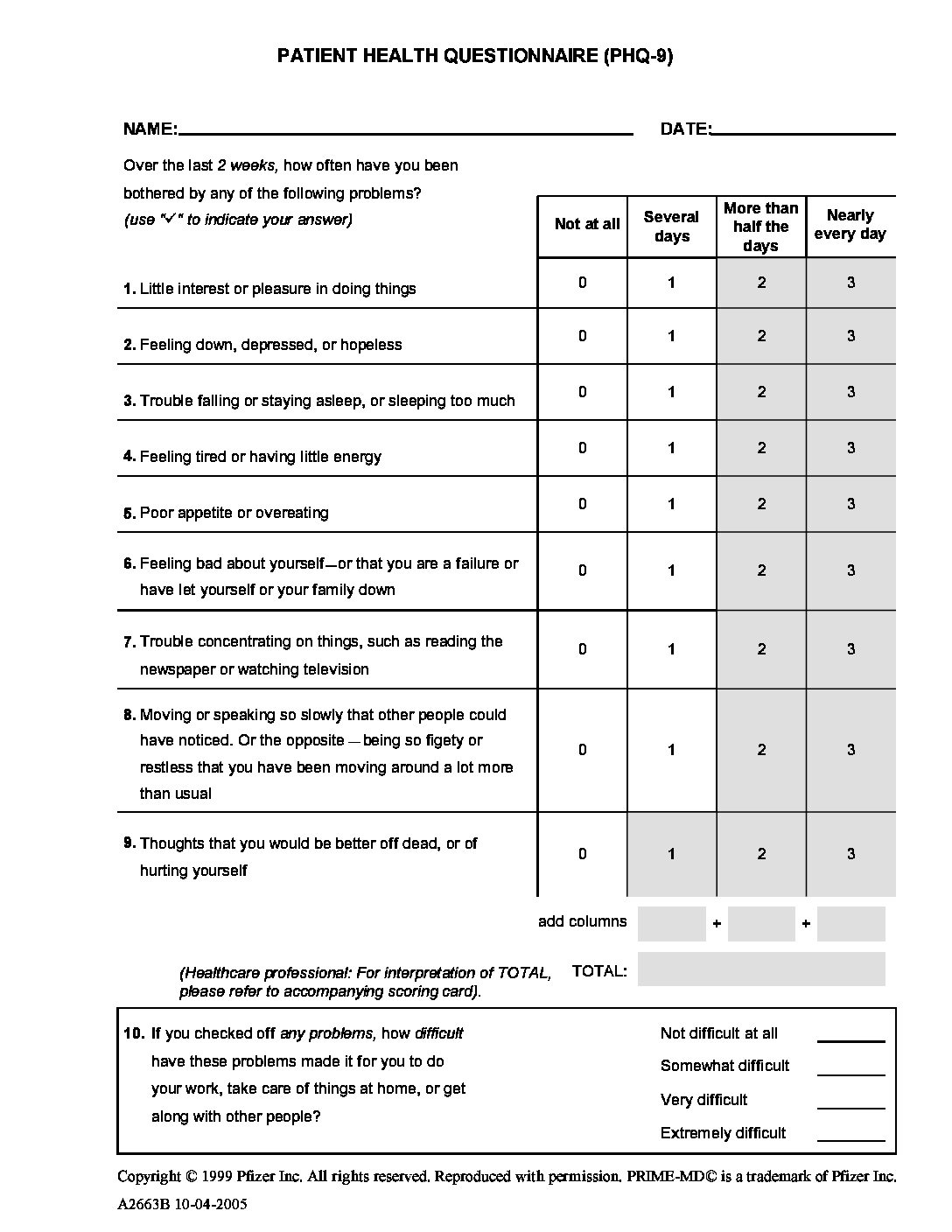


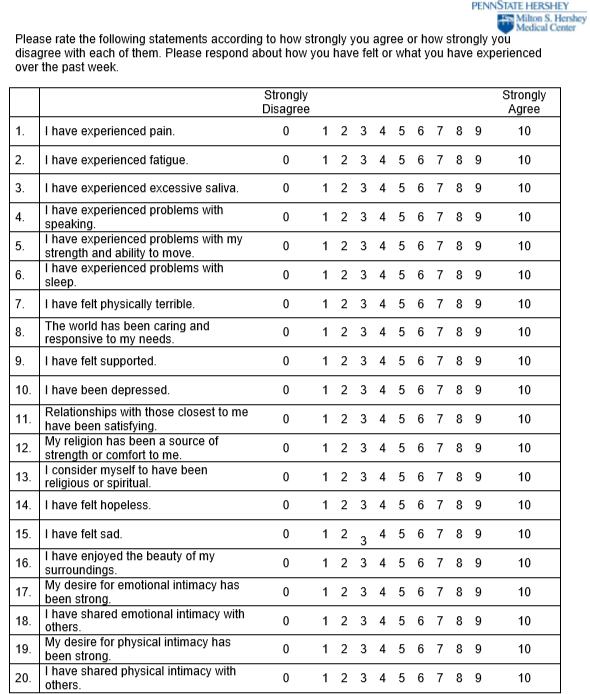


| 1 | Would you say that in general your health is excellent, very good, good, fair, or poor? |  |
| --- | --- | --- |
| 2 | Now thinking about your physical health, which includes physical illness or injury, for how many days during the past 30 days was your physical health not good? |  |
| 3 | Now thinking about your mental health, which includes stress, depression and problems with emotions, for how many days during the past 30 days was your mental health not good? |  |
| 4 | During the past 30 days, for about how many days did poor physical or mental health keep you from doing your usual activities, such as self-care, work or recreation?> |  |


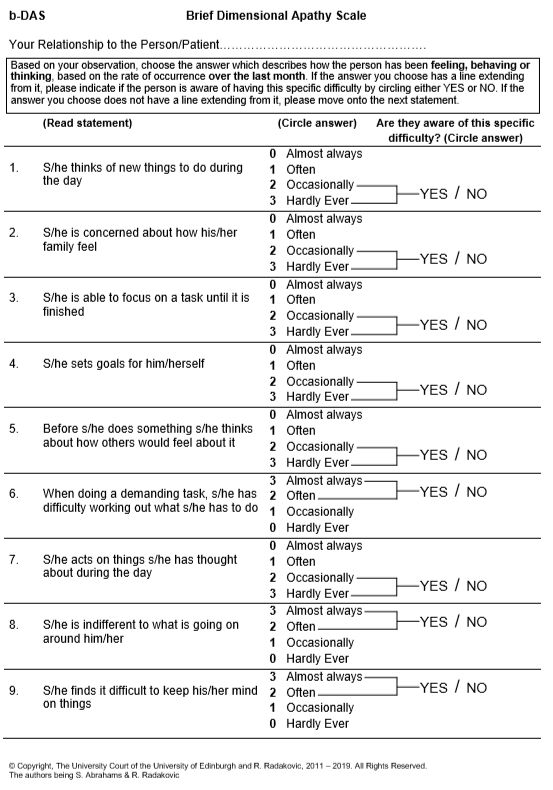

Supplement: Supplementary file 2 — Supplementary file2 (DOCX 908 KB) [file 415_2023_12010_MOESM2_ESM.docx]
